# Metachronous rupture of a residual pancreaticoduodenal aneurysm after release of the median arcuate ligament: a case report

**DOI:** 10.1186/s40792-020-0784-5

**Published:** 2020-02-03

**Authors:** Nana Kimura, Koshi Matsui, Kazuto Shibuya, Isaku Yoshioka, Norihito Naruto, Yui Hoshino, Kosuke Mori, Katsuhisa Hirano, Toru Watanabe, Shozo Hojo, Shigeaki Sawada, Tomoyuki Okumura, Takuya Nagata, Kyo Noguchi, Tsutomu Fujii

**Affiliations:** 1 Department of Surgery and Science, Graduate School of Medicine and Pharmaceutical Sciences, University of Toyama, 2630, Sugitani, Toyama, 930-0194 Japan; 2 Department of Radiology, Graduate School of Medicine and Pharmaceutical Science, University of Toyama, 2630, Sugitani, Toyama, 930-0194 Japan

**Keywords:** Median arcuate ligament syndrome, Multiple aneurysm, Metachronous rupture, Segmental arterial mediolysis

## Abstract

**Background:**

Multiple pancreaticoduodenal artery aneurysms in association with median arcuate ligament syndrome (MALS) are relatively rare. A treatment option, such as a median arcuate ligament (MAL) release or embolization of the aneurysms, should be considered in such cases, but the treatment criteria remain unclear.

**Case report:**

A 75-year-old man was transferred to our hospital because of a ruptured pancreaticoduodenal aneurysm. Emergency angiography showed stenosis of the root of the celiac axis (CA), a ruptured aneurysm of the posterior inferior pancreaticoduodenal artery (PIPDA), and an unruptured aneurysm of the anterior inferior pancreaticoduodenal artery (AIPDA). Coil embolization of the PIPDA was performed. Five days after embolization, the gallbladder became necrotic due to decreased blood flow in the CA region, and an emergency operation was performed. We performed a cholecystectomy and released the MAL to normalize the blood flow of the CA region. However, the patient died on postoperative day 8 because of rupture of the untreated aneurysm of the AIPDA.

**Conclusions:**

This is the first report of metachronous ruptures of multiple pancreaticoduodenal aneurysms due to MALS, even after a MAL release. Although rare, a residual aneurysm in the pancreatic head region may need to be embolized quickly.

## Background

Median arcuate ligament syndrome (MALS) is a relatively rare disease in which the median arcuate ligament (MAL) compresses the root of the celiac axis (CA). Chronic compression leads to luminal narrowing of the celiac trunk and reduced blood supply to the abdominal splanchnic organs. To compensate for decreased blood flow in the CA area, blood flow from the superior mesenteric artery to the gastroduodenal artery is usually increased through the inferior pancreaticoduodenal artery, possibly resulting in pseudoaneurysms and spontaneous bleeding along the way, i.e., in the pancreatic head arcade [1]. Coil embolization is performed for aneurysm ruptures. On the other hand, endovascular treatment for CA stenosis or MAL release on laparotomy is performed if an aneurysm is not ruptured [2]. However, precise treatment indications and criteria remain unclear. Herein, we report an unusual case of metachronous ruptures of multiple pancreaticoduodenal aneurysms, even after a MAL release.

## Case presentation

**Fig. 1:**
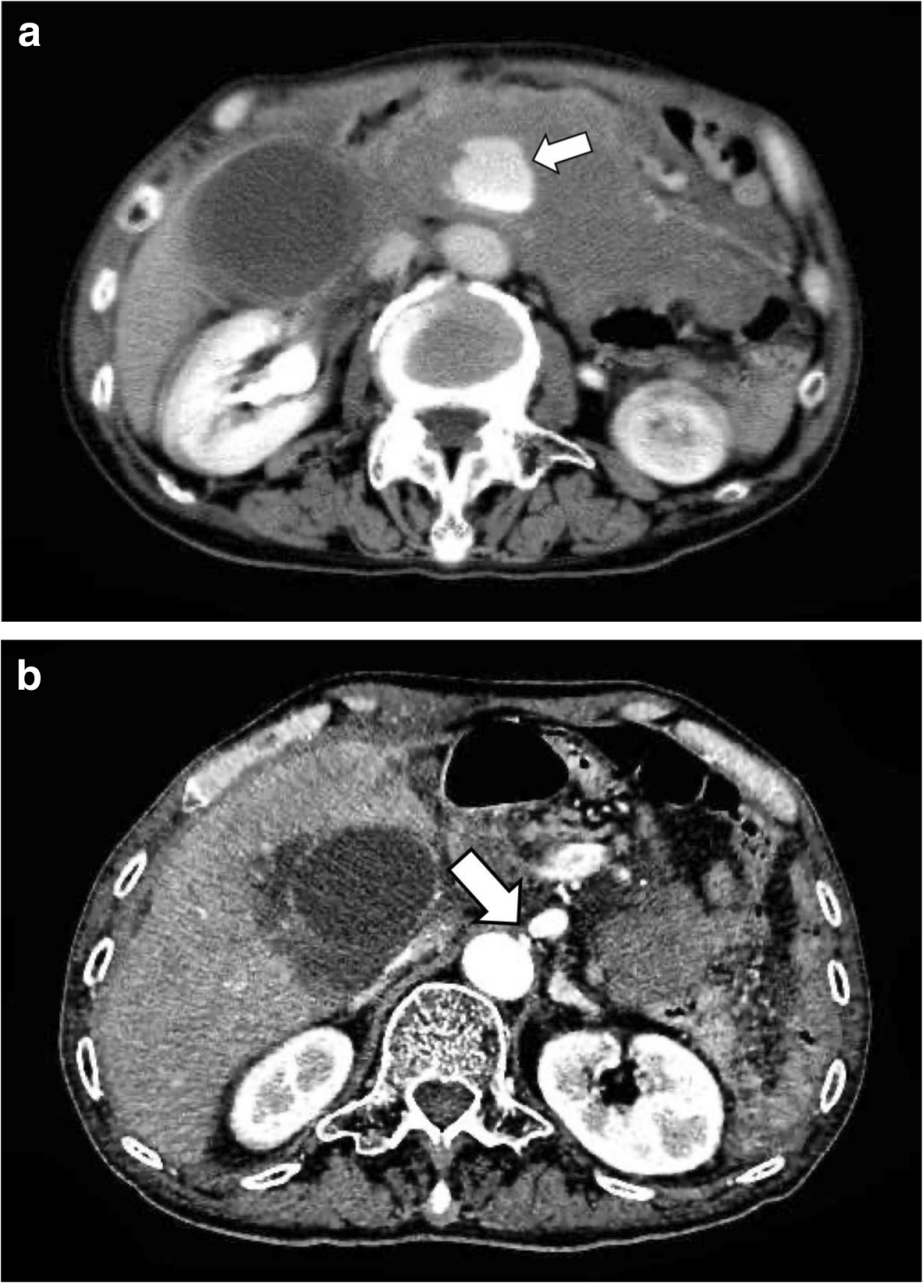
**a** Computed tomography taken at visiting a physician showed a hematoma, and inferior pancreaticoduodenal artery aneurysm rupture was suspected (shown by arrow). **b** Computed tomography taken at visiting a physician showed stenosis of the root of the celiac axis (shown by arrow)

A 75-year-old man visited a physician for abdominal pain and vomiting. Abdominal computed tomography (CT) indicated a ruptured pancreaticoduodenal aneurysm (Fig. 1a) and stenosis of the root of the CA (Fig. 1b). He was transferred to our hospital. He had a past medical history of mental retardation and gastric cancer that had been treated with a distal gastrectomy with Billroth-I reconstruction.

**Fig. 2:**
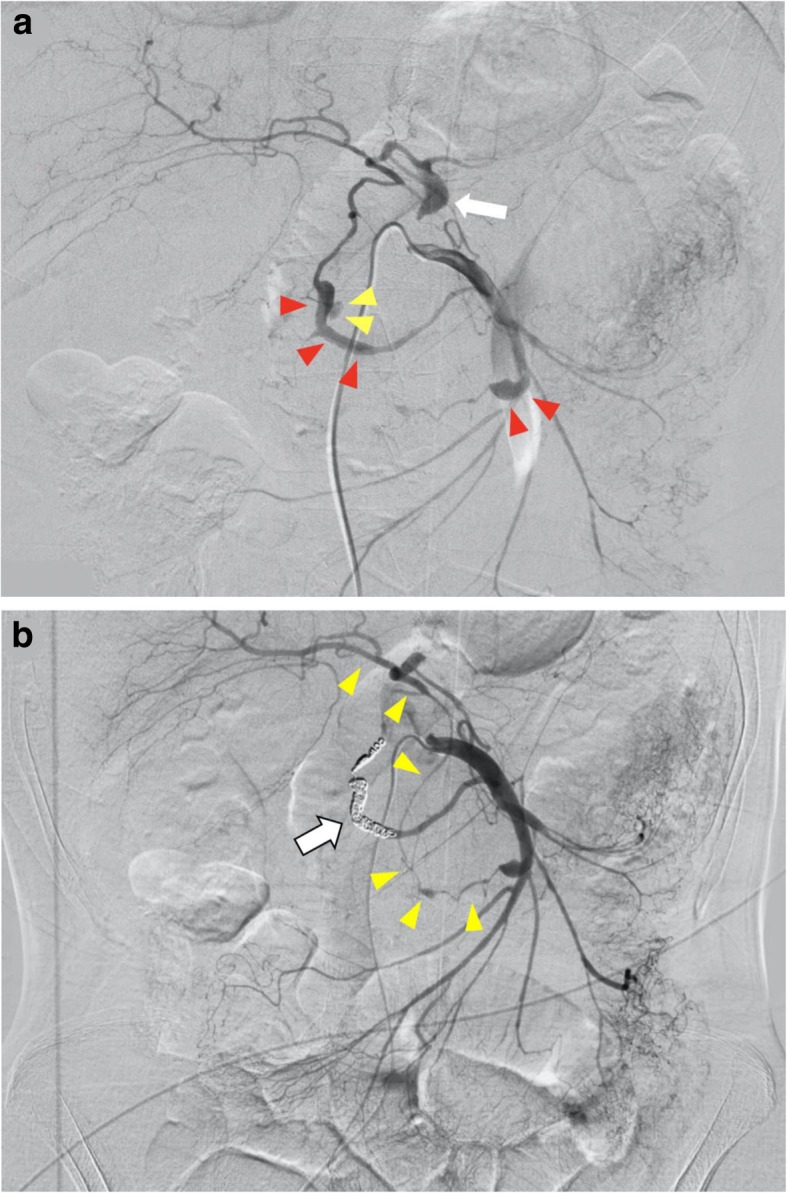
**a** Emergency angiography revealed stenosis of the origin of the celiac artery (shown by arrow), and spindle-shaped dilatation and pseudoaneurysm formation were observed in posterior inferior pancreaticoduodenal artery to posterior superior pancreaticoduodenal artery and anterior inferior pancreaticoduodenal artery (shown by red arrowheads). Contrast medium extravasation from posterior inferior pancreaticoduodenal artery was observed (shown by yellow arrowheads). Irregular vasodilation and stenosis were observed in multiple arteries, which were considered to be the effect of segmental arterial mediolysis. **b** Embolization was performed on posterior inferior pancreaticoduodenal artery to posterior superior pancreaticoduodenal artery (shown by arrow). After coil embolization of the posterior inferior pancreaticoduodenal arterial aneurysm, the celiac arterial region was visualized from the anterior inferior pancreaticoduodenal artery via the gastroduodenal artery (shown by arrowheads)

**Fig. 3:**
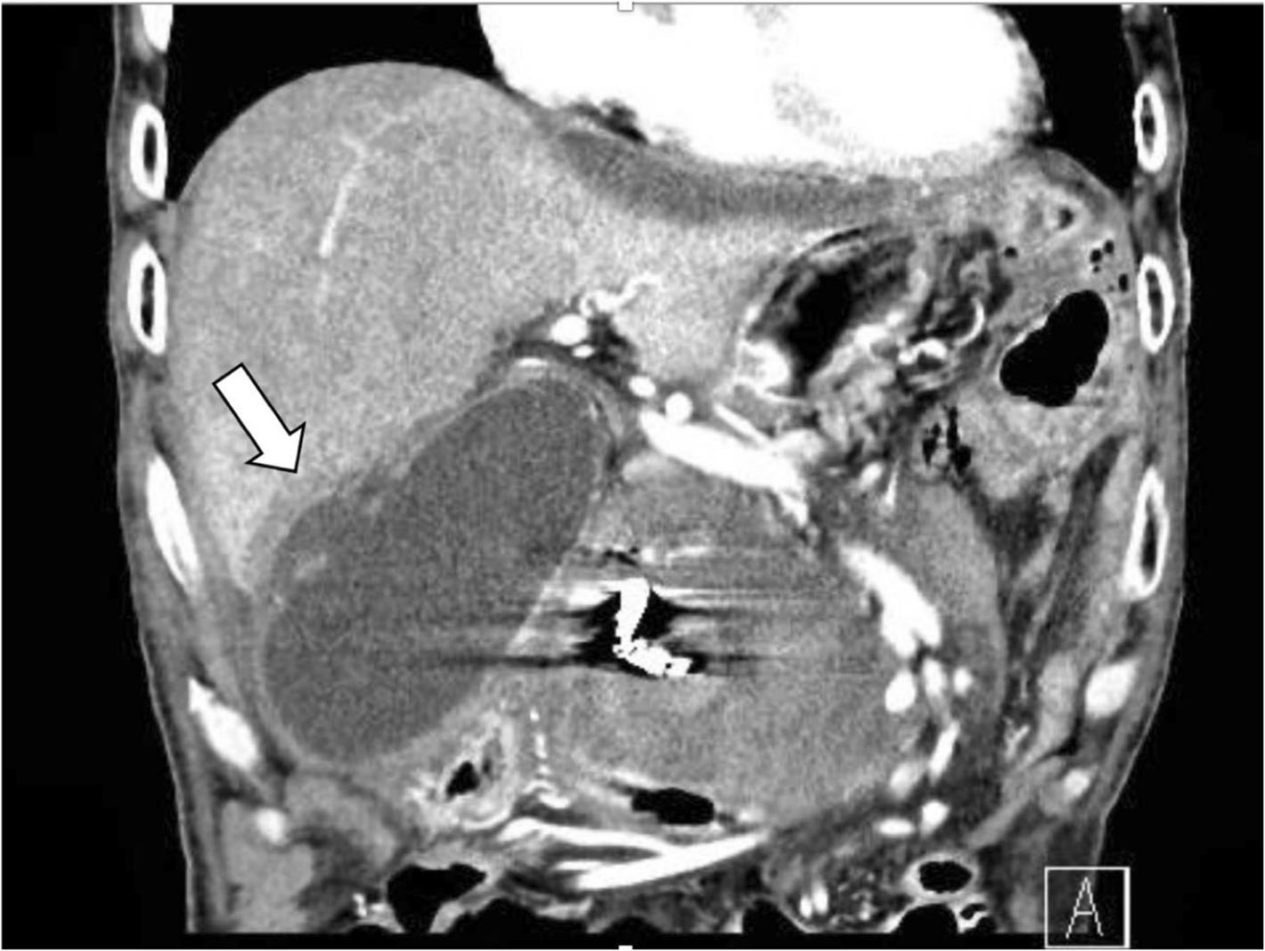
Post-embolization computed tomography shows swollen gallbladder and encapsulated fluid retention around it (shown by arrow). It suggested that the wall was broken at the bottom of the gallbladder

Blood examination findings revealed an elevation of inflammation markers (white blood cell 19,010/μL, C-reactive protein 7.08 mg/dL) and anemia (hemoglobin 8.9 g/dL) (Table 1). Emergency angiography revealed stenosis of the root of the CA, and spindle-shaped dilatation and pseudoaneurysm formation were observed both in the posterior inferior pancreaticoduodenal artery (PIPDA) and in the anterior inferior pancreaticoduodenal artery (AIPDA). Contrast medium extravasation from the PIPDA was observed (Fig. 2a). Coil embolization was performed on the PIPDA to the posterior superior pancreaticoduodenal artery, which was the bleeding source (Fig. 2b). At that time, coil embolization of the AIPDA was not performed because no extravasation of the contrast agent was observed. After coil embolization of the PIPDA, the celiac arterial region was visualized from the anterior inferior pancreaticoduodenal artery via the gastroduodenal artery. Then, the patient was hospitalized for follow-up, but right-sided flank pain appeared on the sixth day after the embolization. CT showed a swollen gallbladder and encapsulated fluid retention around it. It suggested that the wall was broken at the fundus of the gallbladder (Fig. 3).

**Table 1:** Clinical laboratory data on hospital admission

Alb	2.8 g/dL	WBC	19,010 /μL
AST	303 U/L	RBC	345 × 10^4^ /μL
ALT	249 U/L	Hb	8.9 g/dL
T-Bil	2.7 mg/dL	Ht	27.9 %
D-Bil	2.2 mg/dL	PLT	15.6 × 10^4^/μL
LDH	318 U/L	Blood gas	
ALP	535 U/L	pH	7.204
γGTP	176 U/L	HCO3-	15.6 × 10/μL
PT-%	38%	BE	− 17.2 mmol/L
BUN	16 mg/dL	Lac	12.8 mmol/L
CRE	1.07 mg/dL		
CRP	7.08 mg/dL

**Fig. 4:**
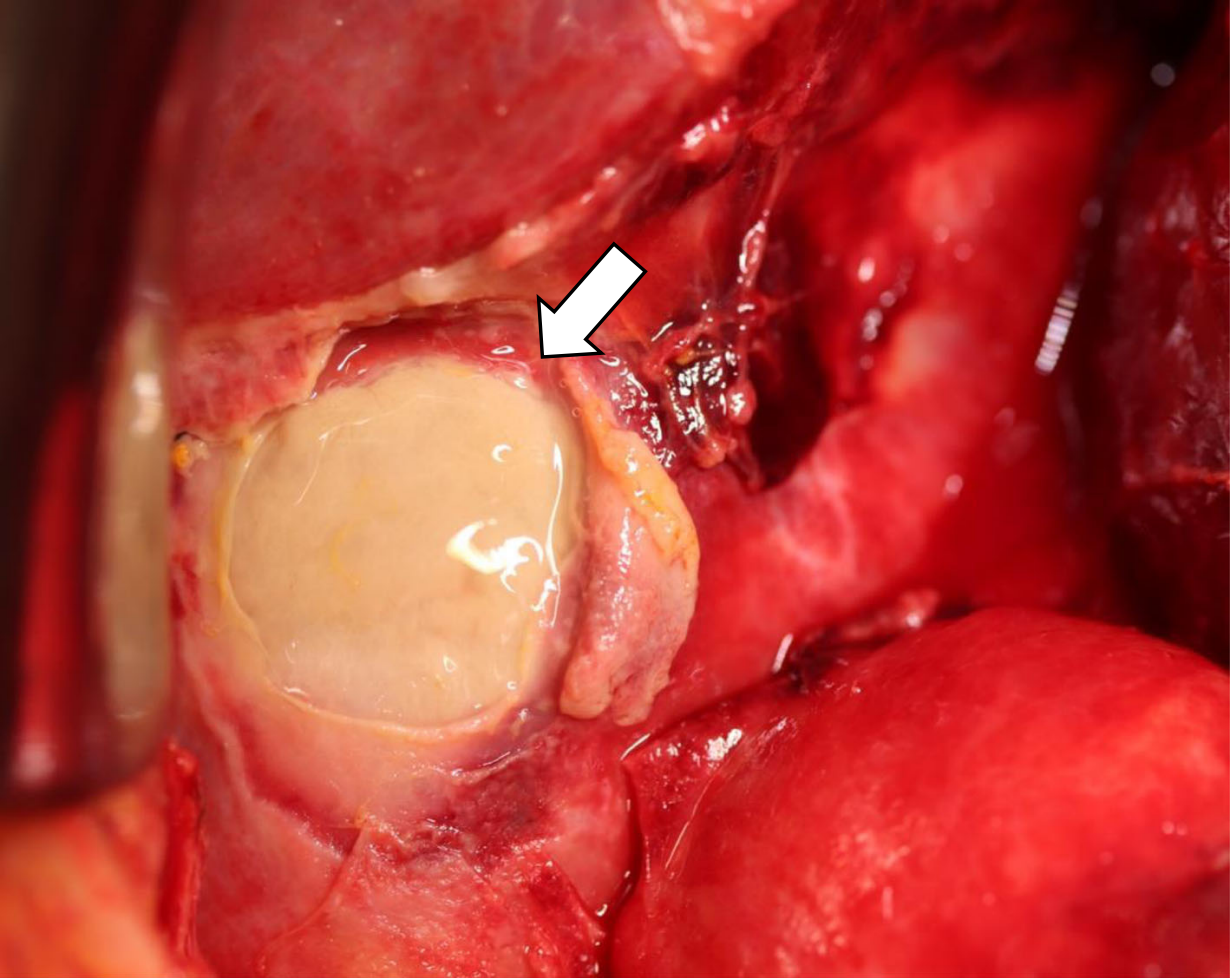
The intraoperative finding showed the wall of the gallbladder was partially necrotic (shown by arrow)

**Fig. 5:**
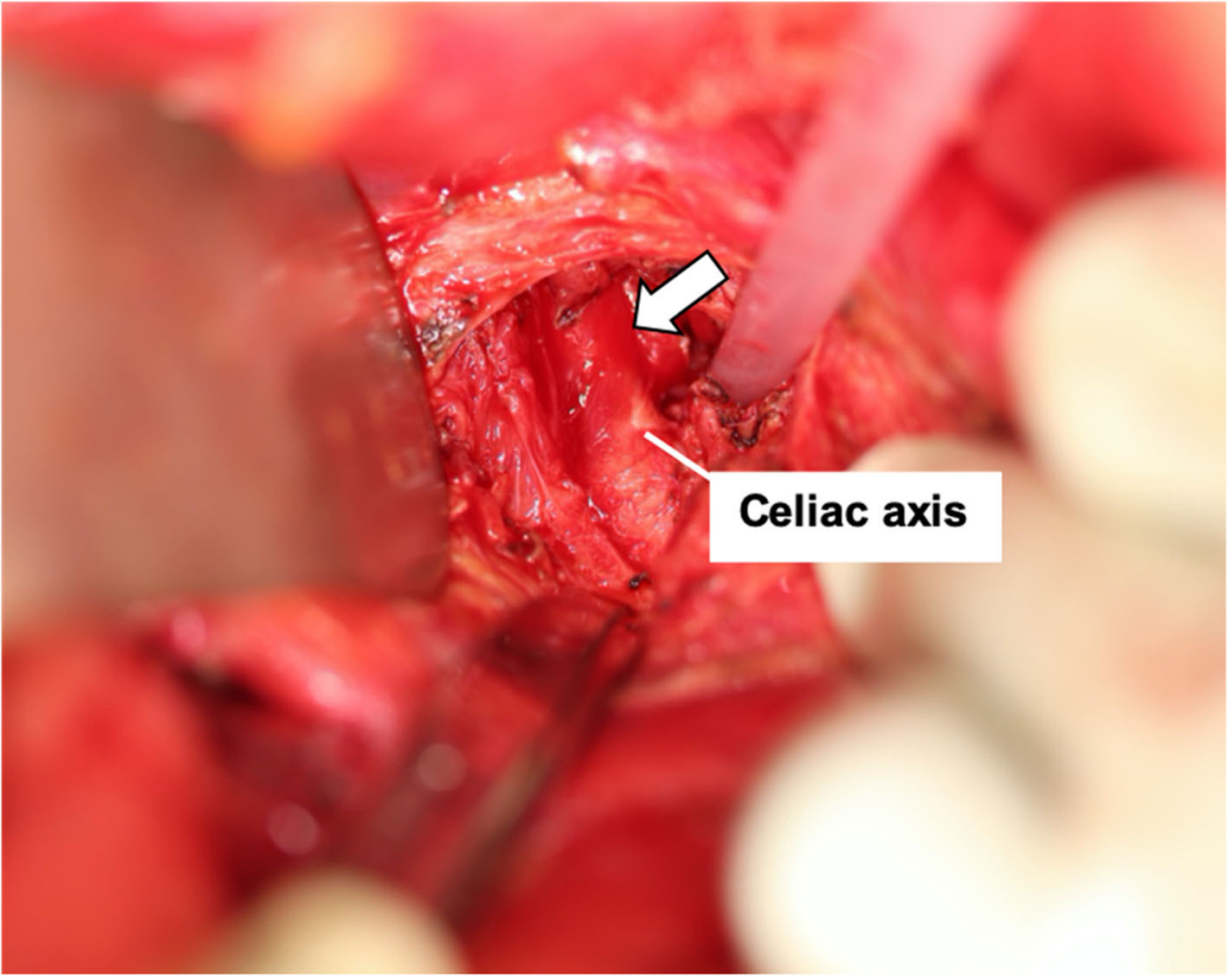
The intraoperative identification of the median arcuate ligament (shown by arrow)

An emergency laparotomy was then performed because gallbladder necrosis was suspected due to the decreased blood flow in the CA region. The wall of the gallbladder was found to be partially necrotic (Fig. 4). The gallbladder was distended, and bile leakage was not observed. In addition, the MAL was dissected to improve blood flow in the CA region. A fibrous ligament anterior to the CA was confirmed, and the MAL was transected to expose the wall of the CA and the aorta (Fig. 5). The blood flow in the celiac and hepatic arteries was confirmed by echography, and the surgery was completed after the abdominal cavity was thoroughly washed.

**Fig. 6:**
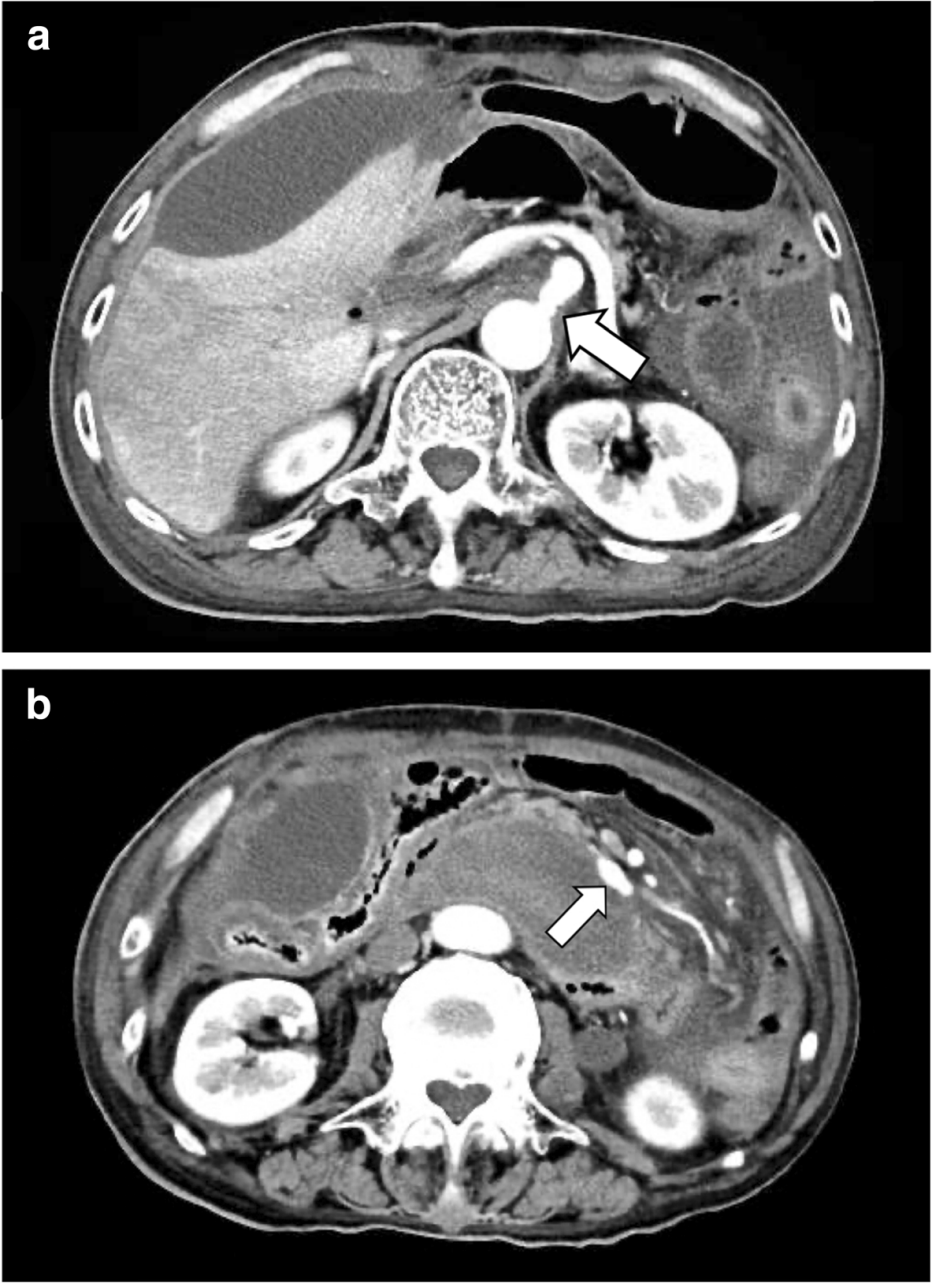
**a** The abdominal computed tomography on postoperative day 4 determined that the arterial diameter of the celiac axis has expanded slightly (shown by arrow). **b** The abdominal computed tomography on postoperative day 4 determined that there was no significant change in the size (13 × 7 mm) of the untreated aneurysm of the anterior inferior pancreaticoduodenal artery (shown by arrow)

**Fig. 7:**
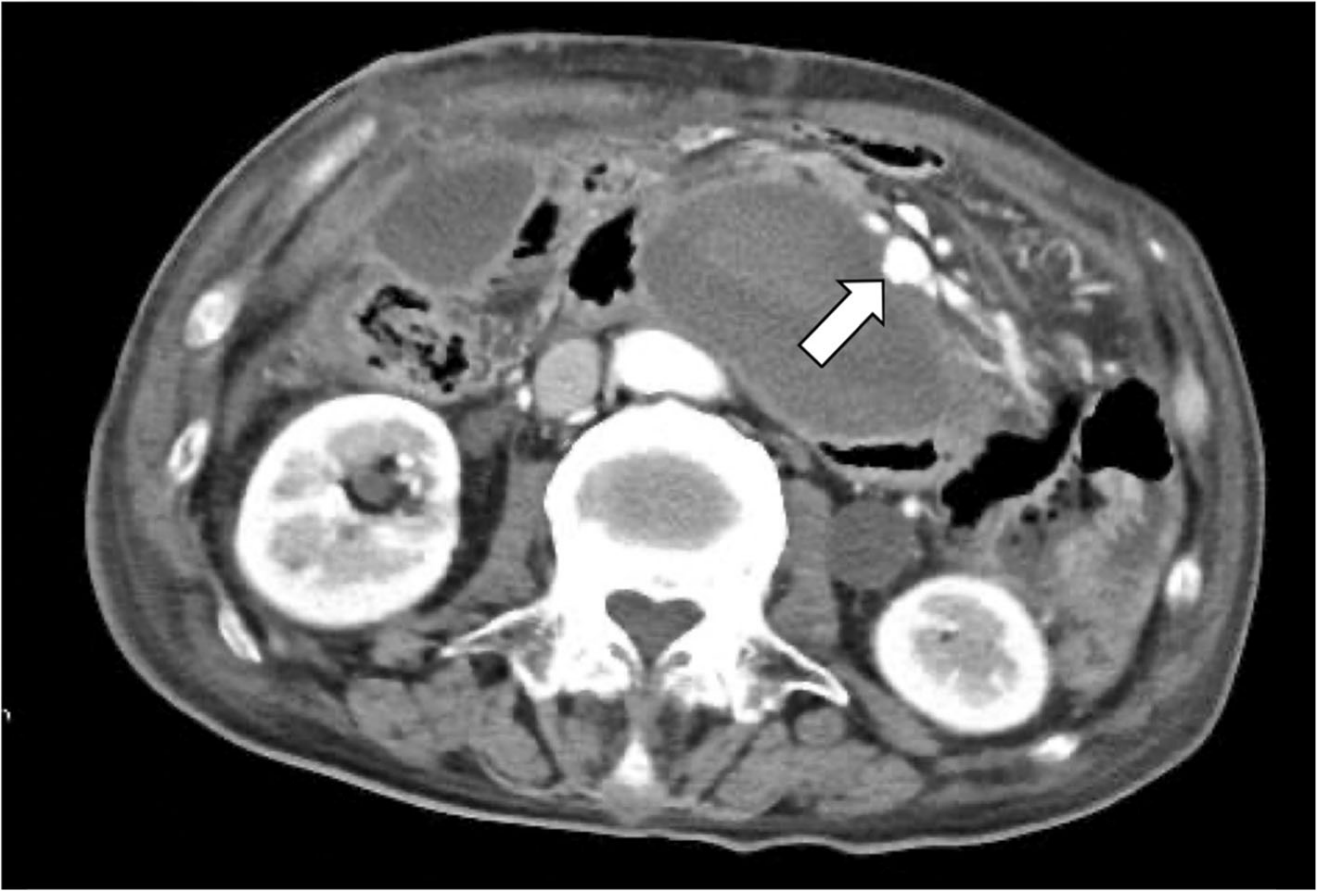
Computed tomography after first vomiting showed extravasations from the untreated anterior inferior pancreaticoduodenal artery aneurysm (shown by arrowheads). An untreated anterior inferior pancreaticoduodenal artery aneurysm appeared to be rounded (13 × 11 mm)

After the operation, the patient entered the emergency care unit under intubation. Extubation was performed on postoperative day (POD) 2. Perioperative blood pressure was controlled with antihypertensive agent so that systolic blood pressure was around 100 mmHg. Pleural effusion and ascites were observed, and bilateral thoracic drainage and ascites drainage were performed on POD 4. The abdominal CT scan on POD 4 determined that the stenosis of the CA was released (Fig. 6a) and that the size of the untreated aneurysm of the AIPDA was unchanged (Fig. 6b). However, the patient exhaled blood on the evening of POD 8, and an emergency CT scan and upper gastrointestinal endoscopy were performed. At that time, no obvious source of bleeding was found, and fresh blood had accumulated in the esophagus. The emergency CT findings showed little change in the peritoneal hematoma and extravasations from the untreated AIPDA aneurysm. In addition, the AIPDA aneurysm, which had been unchanged in the previous CT scan, increased slightly from the previous CT scan (Fig. 7). The patient soon exhaled blood again, and his systolic blood pressure dropped rapidly to less than 60 mmHg. Because of heavy bleeding, endotracheal intubation was performed to control the airway. After transfusion, another upper gastrointestinal endoscopy was performed. Persistent bleeding from the posterior wall of the descending duodenum was observed, and the duodenal wall broke down. We consulted radiology about the indication of endovascular treatment, but it was judged difficult because of vital instability. The patient died of hemorrhagic shock 12 h after the first hematemesis. An autopsy was not performed because the consent of the family was not obtained. Eventually, the rupture of an untreated AIPDA aneurysm was diagnosed as the cause of death.

## Discussion

An abdominal visceral aneurysm is a relatively rare condition. It was reported that 60% of abdominal visceral aneurysms occur in a splenic artery, 20% in a hepatic artery, 10% in the superior mesenteric artery, and 2% in the pancreaticoduodenal artery [3]. A ruptured abdominal visceral aneurysm has a poor prognosis; the mortality rates are 25% for a splenic artery, 35% for a hepatic artery, and 50% for a pancreaticoduodenal artery [3]. Therefore, a rapid and accurate treatment is desirable. The cause of visceral aneurysms varies from arteriosclerosis and inflammation to stenosis. In particular, there are many reports of pancreaticoduodenal aneurysms caused by MALS [4, 5].

MALS is a disorder caused by compression of the root of the CA by the MAL, resulting in decreased blood flow in the CA region [1]. Instead, blood flow in the common hepatic and splenic arteries is compensated for by the superior mesenteric artery via the pancreatic head arcade. Increased blood flow induces hemodynamic stress on the arterial wall of the pancreatic head arcade, causing an aneurysm in the pancreatic head region. In the present case, CA stenosis due to MALS was considered to be the cause of the pancreaticoduodenal aneurysm.

Coil embolization is usually performed for ruptured aneurysms due to MALS [6, 7]. We performed embolization of the ruptured aneurysms in the PIPDA. On the sixth day after the embolization, we performed an emergent laparotomy because the gallbladder became necrotic. In addition to the cholecystectomy, the surgical incision was located in the MAL to release the stenosis of the root of the CA.

There are two types of treatment for MALS that normalize the blood flow in the CA region: endovascular treatment and surgical incision of the MAL [2]. Recently, endovascular treatment has often been selected because of the high risk of surgery [8]. Sugae et al. evaluated CA stenosis due to MAL compression with 3D-CT images and classified them into three types according to the stenosis rate and stenosis length: type A, < 50% and ≤ 3 mm; type B, 50–80% and 3–8 mm; type C, 80–100% and ≥ 8 mm, respectively [9]. Although 3D-CT was not used in the present case, the stenosis was considered type B, and the MAL division was recommended. In the present case, an emergent laparotomy was necessary, so we performed a MAL release at the same time for emergent cholecystectomy to normalize the blood flow of the CA region and to prevent further aneurysm rupture in the pancreatic head arcade arteries. Pancreaticoduodenal aneurysms are different from other abdominal visceral aneurysms because of the low correlation between the diameter of the aneurysm and the possibility of rupture. Fujisawa et al. reported that 41 (71%) out of 58 ruptured pancreaticoduodenal aneurysms are less than 20 mm in diameter [10]. Thus, it is reported that treatment is necessary even if the diameter of the aneurysm is small [11]. There are some reports that a MAL release can be postponed for aneurysms in the pancreatic head region arteries, even for multiple ones [12]. Similarly, in the present case, we thought that a MAL incision would decrease blood flow from the superior mesenteric artery to the pancreatic head arcade and reduce the risk of rupture of the untreated aneurysms. There are many reports that the long-term prognosis after a MAL release is good [13, 14]. There is also a report that among multiple aneurysms, large aneurysms were embolized in the inferior pancreaticoduodenal artery, but small ones were left untreated, and the MAL was not incised but did not rupture [15]. Unfortunately, untreated aneurysms were lethally ruptured even after a MAL release in the present case.

This case is characterized by an aneurysm at the root of the AIPDA in addition to the PIPDA, as shown by an angiography at the time of admission. Multiple pancreaticoduodenal aneurysms have rarely been reported in the international English language literature. Possible causes of aneurysms in multiple arterial systems include arteriosclerosis, pancreatitis, trauma, congenital malformation, fibromuscular hyperplasia, infection, collagen disease, and segmental arterial mediolysis (SAM) [3, 10, 16]. SAM is a concept proposed by Slavin and Gonzalez-Vitale in 1976 and is a noninflammatory and non-arteriosclerotic degenerative disease of uncertain cause that occurs in arteries [16]. It is an acute disease that is subject to emergency treatment, mainly due to medial lysis of the muscular arteries in the abdominal organs, formation of an aneurysm, and rupture of the abdominal cavity. A definitive diagnosis of SAM requires a biopsy of the affected artery and pathologic evaluation [17]. Although there is a possibility that SAM coexisted with MALS in this case, there are few similar reports [18]. Moreover, no pathological finding could be obtained, and in general, it has been reported that SAM can be conservatively treated if it does not bleed or rupture [19]. There are also reports of complete disappearance of SAM with conservative treatment [20].

We did not choose the option to embolize the untreated aneurysm immediately after the MAL release. However, in this case, another aneurysm fatally ruptured despite MAL release and normalized blood flow. It may have been necessary to embolize an untreated AIPDA aneurysm immediately after the release of the MAL. There are no reports of early aneurysm rupture after MAL release, as occurred in this case. Multiple aneurysms caused by MALS may require attention to another aneurysm rupture, even after a MAL release, if one ruptures. The rare clinical course of this case may make a valuable contribution to the development of treatment strategies in the future.

## Conclusions

This is the first report of metachronous ruptures of multiple pancreaticoduodenal aneurysms due to MALS, even after a MAL release. Although rare, a residual aneurysm in the pancreatic head region may need to be embolized quickly.

## Data Availability

The dataset supporting the conclusion of this article is included within the article.

## Abbreviations

AIPDA: Anterior inferior pancreaticoduodenal artery
CA: Celiac axis
CT: Computed tomography
MAL: Median arcuate ligament
MALS: Median arcuate ligament syndrome
PIPDA: Posterior inferior pancreaticoduodenal artery
POD: Postoperative day
SAM: Segmental arterial mediolysis
